# Integrated analysis of mRNAs and lncRNAs reveals candidate marker genes and potential hub lncRNAs associated with growth regulation of the Pacific Oyster, *Crassostrea gigas*

**DOI:** 10.1186/s12864-023-09543-7

**Published:** 2023-08-10

**Authors:** Yongjing Li, Ben Yang, Chenyu Shi, Ying Tan, Liting Ren, Ahmed Mokrani, Qi Li, Shikai Liu

**Affiliations:** Key Laboratory of Mariculture (Ocean University of China), Ministry of Education, and College of Fisheries, Ocean University of China, Qingdao, 266003 China

**Keywords:** *Crassostrea gigas*, RNA-Seq, Marker genes, *cis*-/*trans*-acting lncRNAs, Growth regulation

## Abstract

**Background:**

The Pacific oyster, *Crassostrea gigas*, is an economically important shellfish around the world. Great efforts have been made to improve its growth rate through genetic breeding. However, the candidate marker genes, pathways, and potential lncRNAs involved in oyster growth regulation remain largely unknown. To identify genes, lncRNAs, and pathways involved in growth regulation, *C. gigas* spat was cultured at a low temperature (15 ℃) to yield a growth-inhibited model, which was used to conduct comparative transcriptome analysis with spat cultured at normal temperature (25 ℃).

**Results:**

In total, 8627 differentially expressed genes (DEGs) and 1072 differentially expressed lncRNAs (DELs) were identified between the normal-growth oysters (cultured at 25 ℃, hereinafter referred to as NG) and slow-growth oysters (cultured at 15 ℃, hereinafter referred to as SG). Functional enrichment analysis showed that these DEGs were mostly enriched in the AMPK signaling pathway, MAPK signaling pathway, insulin signaling pathway, autophagy, apoptosis, calcium signaling pathway, and endocytosis process. LncRNAs analysis identified 265 *cis*-acting pairs and 618 *trans*-acting pairs that might participate in oyster growth regulation. The expression levels of *LNC_001270*, *LNC_003322*, *LNC_011563*, *LNC_006260*, and *LNC_012905* were inducible to the culture temperature and food abundance. These lncRNAs were located at the antisense, upstream, or downstream of the *SREBP1*/*p62*, *CDC42*, *CaM*, *FAS*, and *PIK3CA* genes, respectively. Furthermore, the expression of the *trans*-acting lncRNAs, including *XR_9000022.2*, *LNC_008019*, *LNC_015817*, *LNC_000838*, *LNC_00839*, *LNC_011859*, *LNC_007294*, *LNC_006429*, *XR_002198885.1*, and *XR_902224.2* was also significantly associated with the expression of genes enriched in AMPK signaling pathway, insulin signaling pathway, autophagy, apoptosis, calcium signaling pathway, and endocytosis process.

**Conclusions:**

In this study, we identified the critical growth-related genes and lncRNAs that could be utilized as candidate markers to illustrate the molecular mechanisms underlying the growth regulation of Pacific oysters.

**Supplementary Information:**

The online version contains supplementary material available at 10.1186/s12864-023-09543-7.

## Background

Growth is one of the most important production traits of aquaculture species [1]. Growth is a complex biological trait that is affected by many endogenous and exogenous factors [2–4]. For marine invertebrates such as oysters, growth is dramatically affected by environmental factors. The Pacific oyster (*Crassostrea gigas*) has been widely cultivated around the world because of its wide thermophilic range from 5 to 35 °C. The growth of oysters was increased rapidly between 20 ~ 25 °C but is suppressed under 15 ℃ [5–7]. The nutrient accumulation of oysters varies seasonally, with high glycogen storage in early spring or winter and high lipid synthesis in summer [8]. As a result, oyster growth displays seasonal variations with the fluctuation of water temperature, food availability, and other abiotic factors [9]. However, the internal regulatory elements involved in the growth regulation of the Pacific oysters are largely unknown and warrant further investigation.

For a long time, improving the growth rate of aquaculture species was regarded as the top priority for aquaculture genetic improvement. Since the 1980s, transgenic technology has received increasing attention for the genetic improvement of farmed fish. The human growth hormone (GH) gene has been used to create transgenic fish, including carp, salmon, and tilapia, with rapid growth rates [10]. Shellfish genetic breeding efforts have been made to primarily focus on improvement of economic traits including body weight, growth rate, and survival rate through successive artificial selection over 1~5 generations based on family and mass selection [11, 12]. However, these traditional breeding techniques require a significant amount of labor and material resources, so identifying the potential growth-regulating marker genes will improve the efficiency of breeding efforts. In order to reveal the phenotypic variation of growth traits in the Pacific oyster, molecular genetics approaches have been used to discover numerous microsatellite markers, SNPs, and construct several sets of genetic linkages maps [13–15]. However, the critical genes and pathways involved in oyster growth regulation remain largely unknown.

Identifying growth-related candidate marker genes is essential for a better understanding of growth regulation mechanism and for enhancing efficiency of selective breeding in oysters. Transcriptomics techniques have been used to identify the candidate growth-related genes, pathways, and lncRNAs in many organisms [16–20]. Previously, we discovered that microtubule movement, as well as nucleotide and protein biosynthesis, were potentially important for oyster growth by comparative transcriptome analysis of the fast-growing “Haida No.1” oyster and wild oysters [21]. Recently, lncRNAs have been identified to participate in oyster shell pigmentation [22, 23], gametogenesis and reproduction [24], shell biomineralization [25], environmental temperature adaption [26], and glycogen accumulation [27]. However, the candidate genes and lncRNAs that regulate the growth of *C. gigas* have not been investigated. Genes that regulate growth may in turn be controlled by hub lncRNAs, therefore, identifying these candidate lncRNAs may enable the identification of the candidate marker genes that are responsible for oyster growth control.

This study aims to identify genes, lncRNA, and important pathways that are potentially involved in oyster growth regulation through the comparative transcriptome analysis of oysters cultured at different temperatures. On this basis, we hope to reveal the molecular mechanism of these genes and lncRNAs participating in oyster growth regulation by detecting their expression patterns at different cultured temperatures and nutrient levels. Our study provides a set of genes and lncRNAs which can be used as marker genes of growth in selective breeding programs of the Pacific oyster.

## Results

### Summary of the RNA sequencing data

This RNA sequencing produced 126,331,148, 120,631,402, 132,996,112, 118,341,886, 157,756,806, and 132,302,420 paired-end raw reads from the six samples, respectively. After filtering the low-quality reads, a total of 123,643,702, 118,207,158, 129,358,222, 114,518,344, 134,807,224, and 127,225,080 paired-end clean reads were obtained. The percentage of bases with phred values greater than 30 (Q30) was 90.72% ~ 93.06%, and the GC content was 42.43 ~ 46.29%. After mapping the clean reads to the reference genome with HISAT2, a total of 53.99~76.73% of mapped reads were obtained. Of which, 63.88% ~ 76.70% of reads were aligned to protein-coding genes, and 1.31% ~ 1.84% of reads were aligned to the known lncRNAs. Furthermore, reads mapped to rRNA accounted for 3.12%, 1.0%, 3.41%, 3.62%, 7.48%, and 7.45% in the six samples, respectively (Additional file 1: Table S1).

### Identification and characterization of lncRNAs

**Fig. 1:**
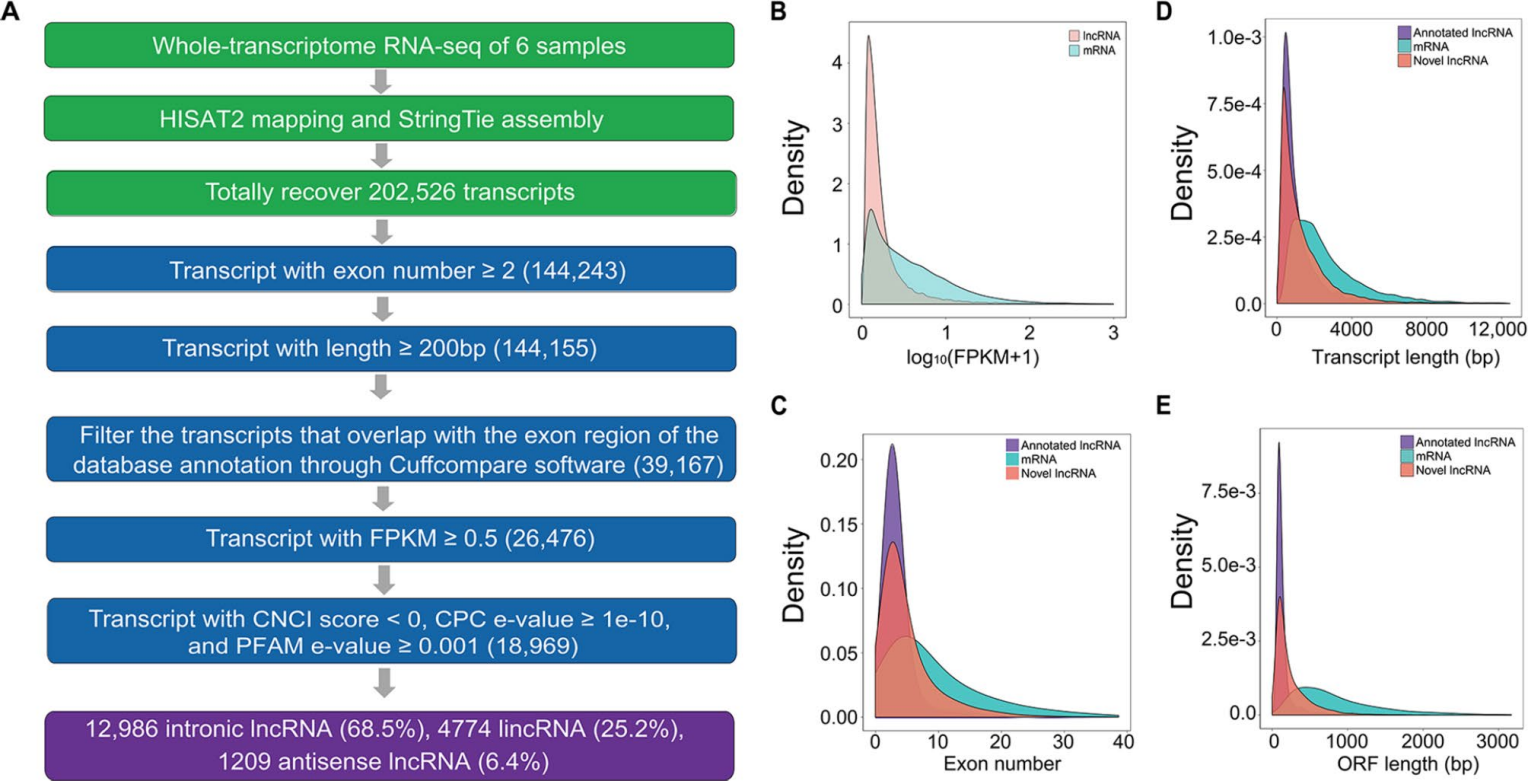
Identification and characterization of lncRNAs. (**A**) An integrative computational pipeline for the systematic identification of the lncRNAs. (**B**) The expression levels of lncRNAs were lower than that of the protein-coding mRNAs. (**C**) The exon number of lncRNAs was smaller than that of mRNAs. (**D**, **E**) The lengths of the transcripts and predicted ORFs were shorter than that of mRNAs

Initial analyses identified 18,969 novel lncRNAs through the integrative computational pipeline as shown in Fig. 1A. The information on the novel lncRNAs and annotated lncRNAs were provided in two separate files (Additional file 2 and Additional file 3). The lncRNAs were classified based on their location in the genome, including intronic lncRNAs (class code “i”), long intergenic noncoding RNAs (lincRNAs, class code “u”), and antisense lncRNAs (class code “x”). Of all the novel lncRNAs, the intronic lncRNAs, lincRNAs, and anti-sense lncRNAs account for 68.5%, 25.2%, and 6.4%, respectively. Expression analysis indicated that the expression levels of lncRNAs were lower than that of the protein-coding mRNAs (Fig. 1B). The gene length, the predicted ORF length, and the exon number were all significantly different between lncRNAs and protein-coding mRNAs. Overall, the exon number of the lncRNAs is smaller than that of the protein-coding mRNAs (Fig. 1C), the lengths of both transcripts and predicted ORFs are shorter in lncRNAs than that of the protein-coding mRNAs (Fig. 1D and E). The features of the novel lncRNAs are similar to the annotated lncRNAs but different from the protein-coding mRNAs.

### Screening potential genes and lncRNAs involved in the oyster growth regulation

The correlation coefficient of gene expression among samples showed that Pearson’s R^2^ values of the six samples were bwtween 0.87 and 0.931. Apparently, higher correlation coefficient was observed between samples within group than that between the two groups, normal-growth oysters and slow-growth oysters (Additional file 4: Fig. S1). Differential expression analysis between the normal- and slow-growth oysters allowed the identification of a total of 8627 DEGs and 1072 DELs (Additional file 1: Table S2 and Table S3). Of these, 3026 DEGs and 202 DELs were expressed significantly higher (*P*-adj < 0.05 and log_2_ FC > 1) in normal-growth oysters, whereas 4701 DEGs and 870 DELs were expressed significantly higher (*P*-adj < 0.05 and log_2_ FC <-1) in slow-growth oysters (Additional file 4: Fig. S2).

### GO and KEGG analysis of the DEGs

**Fig. 2:**
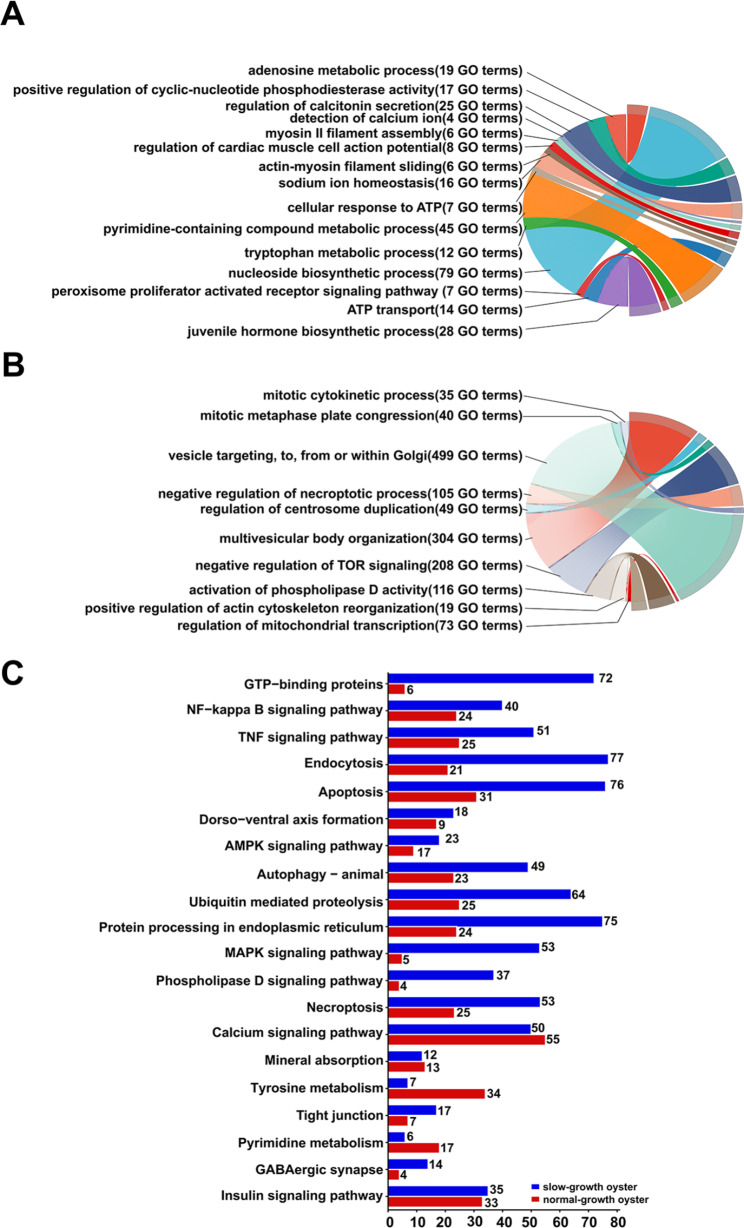
Function analysis of the DEGs in the normal- and slow-growth oysters. (**A**) GO enrichment of the DEGs that were highly expressed in the normal-growth oysters. (**B**) GO enrichment of the DEGs that were highly expressed in the slow-growth oysters. (**C**) KEGG analysis of the DEGs in normal- and slow-growth oysters [75–77]. The blue bar represented the genes highly expressed in slow-growth oysters, and the red bar represented the genes highly expressed in normal-growth oysters

GO terms such as “nucleoside biosynthetic process”, “pyrimidine-containing compound metabolic process”, “sodium ion homeostasis”, and the “tryptophan metabolic processes” were significantly enriched (*P* < 0.05) from the DEGs expressed at higher levels in normal-growth oysters (Fig. 2A, Additional file 1: Table S4). In contrast, the “multivesicular body organization”, “vesicle targeting, to, from or within Golgi”, “negative regulation of TOR signaling”, and several cell proliferation-related processes were significantly enriched (*P* < 0.05) from the DEGs expressed at higher levels in slow-growth oysters (Fig. 2B, Additional file 1: Table S5). KEGG enrichment analysis revealed that the DEGs were significantly enriched (*P* < 0.05) in the pathways related to cell metabolisms and signal transduction, such as the “GTP-binding proteins”, “apoptosis”, “autophagy”, “endocytosis”, “pyrimidine metabolism”, “AMPK signaling pathway”, “insulin signaling pathway”, and “calcium signaling pathways” (Fig. 2C, Additional file 1: Table S6).

### Functional enrichment analysis of the growth-related DEGs

**Fig. 3:**
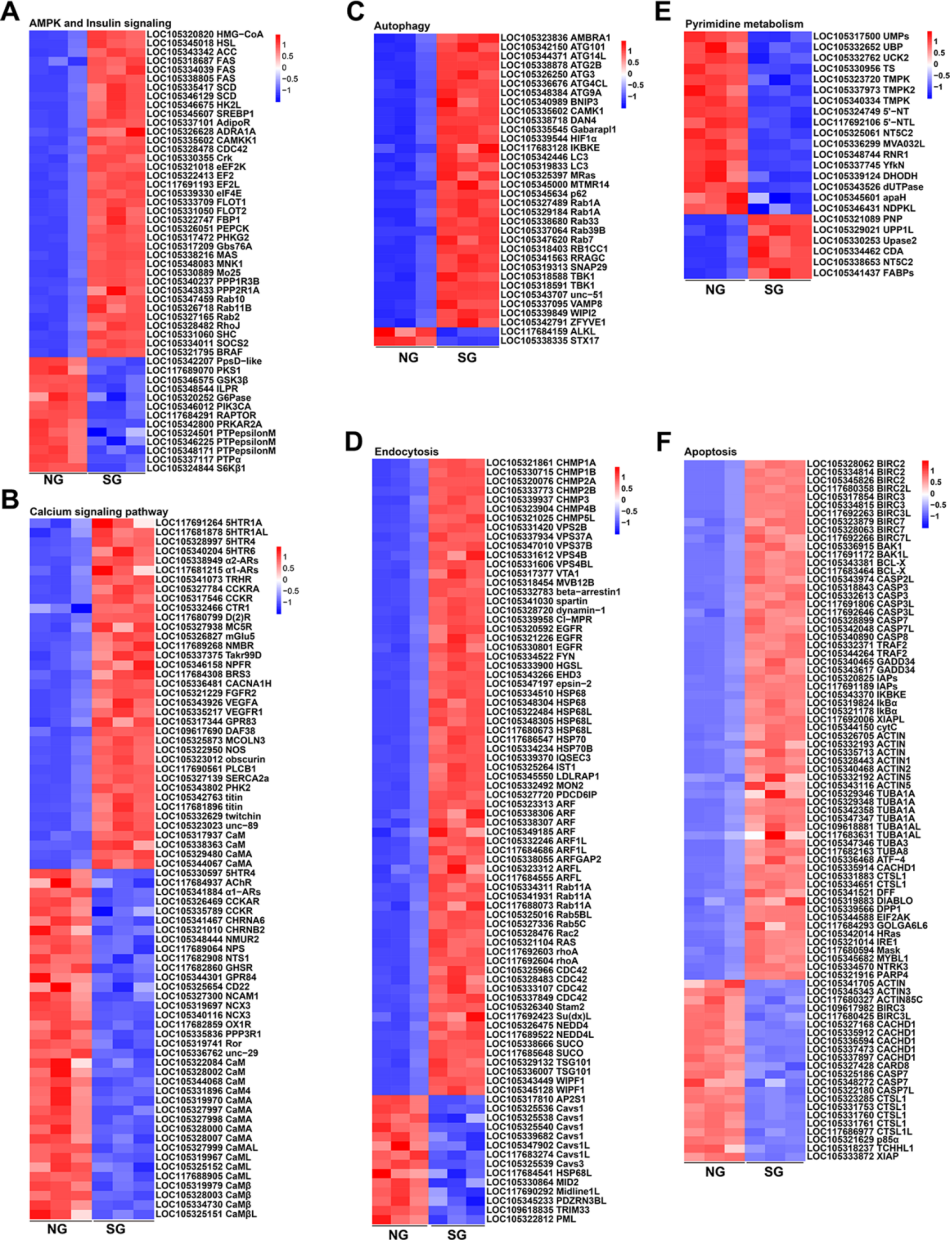
Differentially expressed genes between the normal-growth oyster (NG) and slow-growth oyster (SG). Differentially expressed genes enriched in AMPK and insulin signaling pathways (**A**), calcium signaling pathway (**B**), autophagy (**C**), endocytosis (**D**), pyrimidine metabolism (**E**), and apoptosis (**F**)

As shown in Fig. 3, most of the genes in AMPK signaling, including the *HMG-CoA* (log_2_FC = -2.03), *HSL* (log_2_FC = -2.38), *SCD* (LOC105335417, log_2_FC = -3.56), and *FAS* (LOC105338805, log_2_FC= -3.42) genes were highly expressed in slow-growth (SG) oysters. While the genes in insulin signaling, such as the *ILPR* (log_2_FC = 1.19), *PI3KCA* (log_2_FC = 2.68), *S6Kβ1* (log_2_FC = 2.55), and *RAPTOR* (log_2_FC = 2.45) genes were expressed at a higher level in normal-growth (NG) oysters (Fig. 3A, Additional file 1: Table S7). In addition, 17 *CaM* genes in the calcium signaling pathway were highly expressed in normal-growth (NG) oysters with the log_2_FC ranging from 1.19 to 2.96. Meanwhile, neuropeptide receptors such as the *5HTR4* (log_2_FC = 1.32), *α1-AR*s (log_2_FC = 1.50), *AChR* (log_2_FC = 2.02), *NMUR2* (log_2_FC = 3.04), and *CCKR* (LOC105326469, log_2_FC = 1.45) were all observed to be expressed significantly higher in normal-growth (NG) oysters (Fig. 3B, Additional file 1: Table S7). Genes enriched in autophagy, including *ATG* (LOC105344371, log_2_FC = -2.64), *LC3* (log_2_FC = -2.23), and *p62* (log_2_FC = -3.36) were also highly expressed in the slow-growth (SG) oysters (Fig. 3C, Additional file 1: Table S7). Several endocytosis-related genes, including seven *HSP* genes, seven *CHMP* genes, and five *VPS* genes all highly expressed in slow-growth (SG) oysters (Fig. 3D, Additional file 1: Table S7). In contrast, genes involved in pyrimidine metabolism expressed at higher levels in the normal-growth (NG) oysters with the log_2_FC ranging from 1.14 to 3.08 (Fig. 3E, Additional file 1: Table S7). As for genes enriched in cell apoptosis, seven *tubulin* genes, eight *actin* genes, ten *BIRC* genes, eight *CASP* genes, *IAPs*, *BCL2L2*, *TRAF2*, and *XIAP* genes were highly expressed in slow-growth (SG) oysters (Fig. 3F, Additional file 1: Table S7).

### LncRNAs involved in post-transcriptional regulation of growth-related genes in oysters

**Fig. 4:**
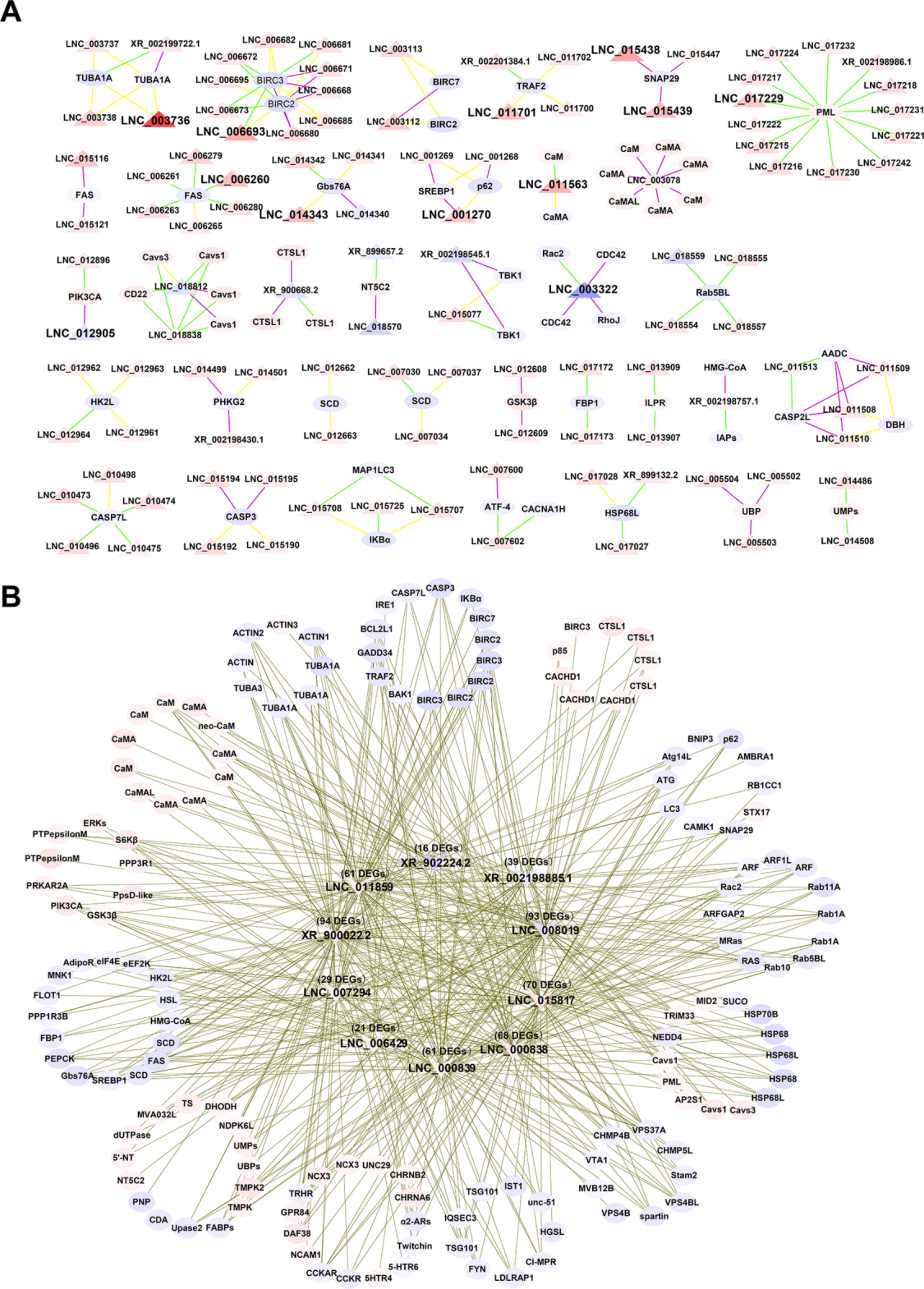
Transcriptional relationship between the DEGs and DELs. (**A**) *Cis*-regulatory networks of DEGs-DELs that participated in the growth regulation. The yellow line represented antisense lncRNAs, the green lines represented lncRNAs located downstream of target genes, and the purple lines represented lncRNAs located upstream of target genes. (**B**) *Trans*-regulatory networks of DEGs-DELs that participated in the growth regulation. The pink represented genes highly expressed in normal-growth oysters, and the blue represented genes highly expressed in slow-growth oysters

The DEGs and their co-localized DELs were used for further *cis*-acting regulation analysis. The 202 DELs were associated with 141 DEGs to form a total of 265 DEG-DEL *cis*-regulatory pairs. Of which, 67 DELs were located on the antisense strand of their target genes, 94 DELs were located upstream of their target genes, and 103 DELs were located downstream of their target genes (Additional file 1: Table S8). Specifically, the DELs highly expressed in normal-growth oysters were found to be involved in the regulation of genes related to apoptosis (e.g. *TUBA1A*, *BIRC3*, *BIRC2*, *TRAF2*) and energy metabolism (e.g. *FAS*, *Gbs76A*). While the DELs highly expressed in the slow-growth oysters were found to be involved in the regulation of genes including the *TBK1*, *PIK3CA*, *Cavs1*, *Cavs3*, *CTSL1*, and *NT5C2* (Table 1). Moreover, other genes related to energy metabolism (e.g. *HMG-CoA*, *SCD*, *PHK*, *PI3K3CA*, *ILPR*, *GSK3β*, *HK2L*), pyrimidine metabolism (e.g. *UMPs*, *NT5C2*, *DHODH*), and endocytosis (e.g. *PML*, *VPS4BL*, *Rab5BL*) were all regulated by DELs, with most *cis*-acting lncRNAs being participated in negative regulation of their target genes (Fig. 4A). According to expression of the DEGs and DELs, a total of 618 DEGs-DELs *trans*-regulatory pairs were discovered, with 546 DEGs-DELs pairs showing similar expression patterns and 72 DEGs-DELs pairs showing opposite expression patterns (Additional file 1: Table S9). Among all these *trans*-regulatory lncRNAs, 10 DELs, including *XR_9000022.2*, *LNC_008019*, *LNC_015817*, *LNC_000838*, *LNC_00839*, *LNC_011859*, *LNC_007294*, *LNC_006429*, *XR_002198885.1*, and *XR_902224.2*, were involved in regulation of 94 DEGs, 93 DEGs, 70 DEGs, 68 DEGs, 61 DEGs, 61 DEGs, 39 DEGs, 29 DEGs, 21 DEGs, and 16 DEGs, respectively. These DEGs and DELs formed a total of 552 DEGs-DELs *trans*-regulatory pairs, which were significantly enriched in the AMPK signaling pathway, insulin signaling pathway, autophagy, apoptosis, calcium signaling pathway, and endocytosis process (Fig. 4B, Additional file 1: Table S9).

**Table 1:** List of DEGs-DELs pairs in normal- and slow-growth oysters

Gene	Gene ID	log_2_FC	Transcript ID	log_2_FC	Location
*TUBA1A*	LOC105329346	-3.70017275	LNC_003736	23.93662779	antisense
*SNAP29*	LOC105319313	-2.4407057	LNC_015438	11.31574412	upstream
*SNAP29*	LOC105319313	-2.4407057	LNC_015439	8.618475833	upstream
*BIRC3*	LOC105334815	-4.30603124	LNC_006693	10.75063802	downstream
*BIRC2*	LOC105334814	-4.33530965	LNC_006693	10.75063802	antisense
*CaM*	LOC105344068	1.461408423	LNC_011563	9.617610056	downstream
*FAS*	LOC105334039	-2.55145345	LNC_006260	7.966093433	downstream
*Gbs76A*	LOC105317209	-3.49655003	LNC_014343	7.90479588	antisense
*Gbs76A*	LOC105317209	-3.49655003	LNC_014340	-2.92424398	upstream
*TRAF2*	LOC105344264	-3.1768215	LNC_011701	7.72984891	antisense
*PML*	LOC105322812	1.535656445	LNC_017229	7.103263228	downstream
*SREBP1*	LOC105345607	-1.83649893	LNC_001270	6.719835127	upstream
*p62*	LOC105345634	-3.36036485	LNC_001270	6.719835127	antisense
*p62*	LOC105345634	-3.36036485	LNC_001268	1.256274931	upstream
*CDC42*	LOC105328478	-1.19557181	LNC_003322	-12.2225691	upstream
*RhoJ*	LOC105328482	-1.58162094	LNC_003322	-12.2225691	upstream
*Rac2*	LOC105328476	-2.14562438	LNC_003322	-12.2225691	downstream
*CHRNB2*	LOC105321010	1.394225302	XR_898875.2	-4.89298338	upstream
*Rab5BL*	LOC105325016	-2.78927276	LNC_018559	-4.72237896	downstream
*TBK1*	LOC105318588	-1.48227797	XR_002198545.1	-4.59689076	upstream
*PIK3CA*	LOC105346575	2.680724181	LNC_012905	-3.70560133	upstream
*Cavs1*	LOC105325540	2.932726223	LNC_018812	-3.61576579	downstream
*Cavs3*	LOC105325539	2.380009549	LNC_018812	-3.61576579	antisense
*CTSL1*	LOC105331761	3.031090695	XR_900668.2	-3.61244523	upstream
*NT5C2*	LOC105325061	1.772432811	XR_899657.2	-2.6749522	downstream

### Validation of the regulatory relationship between lncRNAs and their target genes

**Fig. 5:**
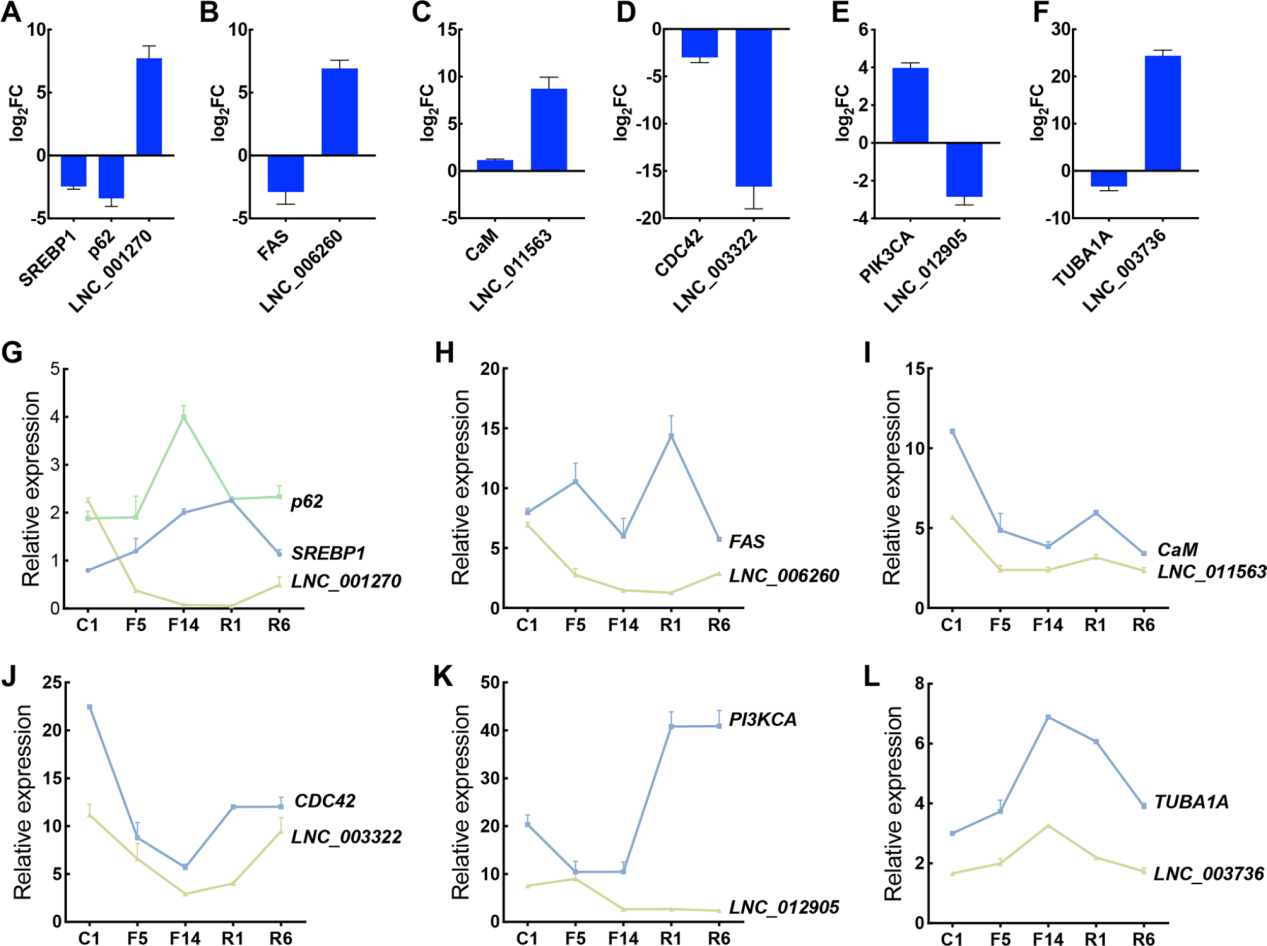
Validation of the regulatory relationship between DEGs and DELs. (**A-F**) The expression patterns of the DEGs-DELs pairs in normal- and slow-growth oysters. Data are presented as means ± SD of the three independent replicates. (**G-L**) The expression patterns of the DEGs-DELs pairs during the fasting and re-feeding process. C represented the control group, F5 and F14 represented 5 and 14 days after fasting treatment; R1 and R6 represented 1 and 6 h after re-feeding. Data are expressed as the mean ± SD (n = 6)

A set of DELs and DEGs with the highest log_2_FC, including *SREBP1*/*p62*-*LNC_001270*, *FAS*-*LNC_006260*, *CaM*-*LNC_011563*, *CDC42*-*LNC_003322*, *PIK3CA*-*LNC_012905*, and *TBUA1A*-*LNC_015438*, were selected for validation by using qRT-PCR (Table 1). As shown in Fig. 5, the expression profiles of the DELs-DEGs pairs were consistent with the RNA-seq results. The target genes were under significantly positive or negative regulation of the lncRNAs which were consistent with the target gene prediction results (Fig. 5A - F). Furthermore, in the fasting and re-feeding treatment of the oysters, expression of *LNC_001270* and *LNC_006260* was decreased with fasting treatment (fasting for 5 and 14 days, referred to as F5 and F14) while increased with re-feeding treatment (refeeding after 1 and 6 days, referred to as R1 and R6). Their target genes, including *SREBP1*, *p62*, and *FAS*, showed opposite expression patterns (Fig. 5G, H). Expression of *LNC_011563* and *LNC_003322* was also decreased with fasting treatment and increased after re-feeding, which was consistent with the expression of their target genes including *CaM* and *CDC42* (Fig. 5I, J). The expression of *LNC_012905* was increased with fasting and decreased with re-feeding, which showed an opposite expression pattern in comparison with the expression of the *PI3KCA* gene (Fig. 5K). In contrast, expression of *LNC_003736* was also increased with fasting and decreased with re-feeding, but showed a consistent expression pattern with the *TUBA1A* target gene (Fig. 5L).

## Discussion

Our study revealed that DEGs between the normal- and slow-growth oysters were mainly enriched in cell metabolism (AMPK signaling, insulin signaling pathway, pyrimidine metabolism, and autophagy), apoptosis, necroptosis, and signaling transduction (endocytosis, calcium signaling pathway process) pathways. Expression analysis indicated that DEGs-DELs pairs, including *SREBP1*/*p62*-*LNC_001270*, *CDC42*-*LNC_003322*, *CaM*-*LNC_011563*, *FAS*-*LNC_006260*, *PIK3CA*-*LNC_012905*, and *TUBA1A*-*LNC_003736* might play important roles in growth regulation, which deserves further investigation in oysters.

### Cell metabolism provides basic energy supply for oyster growth

**Fig. 6:**
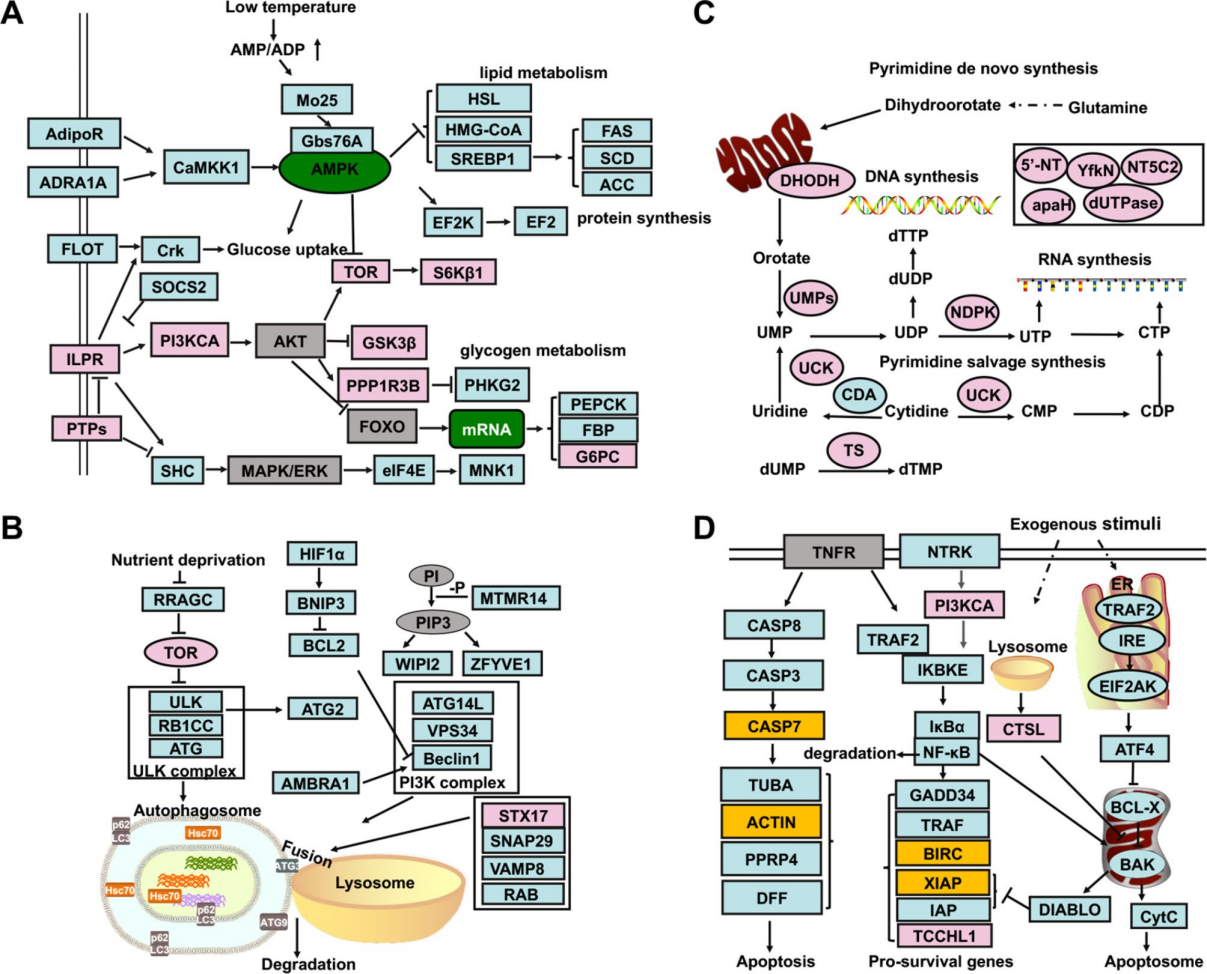
Genes and pathways involved in growth regulation of the oyster. Genes were enriched in signal transduction of AMPK and insulin signaling pathways (**A**), autophagy (**B**), pyrimidine metabolism (**C**), and apoptosis (**D**). The pink represented genes that were highly expressed in normal-growth oysters, the blue represented genes that were highly expressed in slow-growth oysters, the gray represented unchanged genes, the yellow represented the genes that were both highly expressed in the normal- and slow-growth oysters (these genes possessed same gene name but different gene ID), the green represented the key factors in these pathways, the arrows represented a promoting effect and the “-|” represented an inhibiting effect

Under optimal growth conditions, growth factors and their receptor-mediated pathways promote cell proliferation and cell metabolism [28]. In this study, the insulin signaling pathway, ILPR-mediated PI3K/AKT signaling pathway, and TOR signaling pathway were activated to promote oyster growth by regulating glycogen metabolism and protein synthesis, with high expression levels of *ILPR*, *PI3KCA*, *S6Kβ1*, *GSK3β*, and *RAPTOR* being observed in normal-growth (NG) oysters (Fig. 6A). The activity of the insulin receptor depends on several factors. For instance, PTPepsilonM has been shown to play a role in insulin-induced glucose metabolism via direct dephosphorylation and inactivation of IR [29]. Since it is highly expressed in normal-growth oysters, the whole insulin signaling pathway is likely activated in the normal-growth oysters. Furthermore, Ca^2+^ and calmodulin (CaM) contribute to the phosphorylation of the beta subunit of the insulin receptor and regulation of the binding activity of insulin to its receptor. Fluctuations in cytosolic Ca^2+^ concentrations or inhibition of calcium-calmodulin actions are all thought to affect the secretion of peptides and neurotransmitters [30–33]. Highly expressed *CaM* genes in normal-growth oysters might facilitate the activity of insulin signaling and promote the secretion of insulin-like peptides. Current knowledge suggests that biogenic amines, including octopamine (OA), dopamine (DA), and serotonin (5-HT) are all involved in growth regulation by stimulating the release of hormones, such as insulin-like peptides, and activating the process of energy mobilization [34, 35]. Tyrosine metabolism and tryptophan metabolic process/serotonin biosynthetic processes were also increased in normal-growth oysters compared to slow-growth oysters in our study. Tyrosine is an amino acid precursor of norepinephrine and dopamine, and the roles of dopamine and serotonin in regulating the secretion of insulin or insulin-like peptides have been widely studied [36, 37]. All these clues suggest that the insulin-like peptides and the associated factors such as neurotransmitter, kinase, and CaM could work together to control oyster growth by regulating the metabolism activity and the neuroendocrine activity.

Under unfavorable growth conditions, such as nutrient deprivation and temperature stress, AMP-activated protein kinase (AMPK) mediates multiple metabolic processes to replenish cellular ATP supplies. This includes positive regulation of the fatty acid oxidation and autophagy process and negative regulation of ATP-consuming biosynthetic processes including glycogen, lipid, and protein synthesis [38]. In addition, with deficiency of exogenous carbohydrates, gluconeogenesis is activated to provide glucose through utilization of amino acids, lactate, pyruvate, and glycerol. The gluconeogenic enzyme, *PEPCK*, allows hepatic parenchymal cells to produce glucose from pyruvate and other precursors derived from the citric acid cycle [39], while *PHKG2* functions to mediate the glycogenolysis process through phosphorylating and activating glycogen phosphorylase [40]. Both were highly expressed in the slow-growth oysters. Furthermore, the lipid metabolism-related genes such as the *HSL*, S*REBP1*, *FAS*, *ACC*, *SCD*, and *HMG-CoA* genes were all highly expressed in slow-growth oysters, suggesting a need for stimulation of the gluconeogenesis and the fatty acids metabolism activity at low temperature or other growth inhibited condition to support normal survival (Fig. 6A). With high expression levels of the *p62*, *SNAP29*, and *ATG* genes under growth-inhibited conditions (Fig. 6B), the autophagy process was also activated to promote the degradation of macromolecules and organelles in order to maintain the energy balance under the limited nutrient conditions [41]. Furthermore, activation of AMPK under environmental stress is always closely related to the inhibition of PI3K, AKT, and ERK, or inhibition at the level of translation elongation and an increase in autophagy markers. Exposure of cells to ambient stress affects the activity of signaling networks previously implicated in metabolic and growth factor signaling [42]. Our previous study also found that the growth is negatively affected under the experiment temperature (15 ℃) where the food intake activity and metabolism of nutrients were influenced. The genes that are in response to lower temperatures could also be involved in the growth regulation process [43]. Our findings suggest that two important energy-sensing pathways, insulin and AMPK signaling, play critical roles in the modulation of energy supply and consumption to sustain normal oyster growth.

### Cell proliferation, apoptosis, and survival processes are related to oyster growth

During adequate nutrition, growth factors use glucose as energy to promote cell proliferation, but in nutrient-stress conditions, the lack of glycogen not only affects cell metabolism but also affects cell proliferation [44]. Cell division requires adequate amounts of purine and pyrimidine nucleotides for nucleic acid synthesis. Pyrimidine ribonucleotides are essential for DNA and RNA synthesis, cell growth, and proliferation. They provide the necessary building blocks for nucleic acids and precursors for cell membrane synthesis [45]. In this study, the pyrimidine metabolism-related genes were highly expressed under normal growth conditions, indicating that cell proliferation was active (Fig. 6C). Once the nutrients were limited, cells reduced metabolism, went into cell cycle arrest, and eventually induced apoptosis. Apoptosis is an energy-dependent process that eliminates damaged cells and is always accompanied by cytoskeleton rearrangement [46]. The TNF signaling and downstream *caspase 3*, *caspase 7*, *caspase 8*, *actin*, and *tubulin* genes were highly expressed in slow-growth oysters, indicating that the apoptosis process disrupted the stability of the cytoskeleton and inhibited the oyster growth (Fig. 6D). The NF-κB signaling pathway is a key mediator that regulates cell fate in response to various environmental stimuli or growth factors deprivation by driving the up-regulation of proliferative and anti-apoptotic transcripts [47]. In this study, several anti-apoptotic genes, such as *BIRC*, *IAP*s, *XIAP*, and *Bcl2*, were highly expressed in slow-growth oysters and functioned to resist the apoptosis signals induced by adverse environmental conditions (Fig. 6D). Although TNF is a classical pro-inflammatory cytokine, it is also found to be produced from adipose tissue, and associated to obesity-associated metabolic disease [48]. In addition, the crosstalk between TNF and insulin signaling through the transcription factor *GATA6* was revealed in a previous study [49]. The expression of the apoptosis-related protein, ABC transport, GSK-3β, NF-κB, and TOR, are together controlled by the PI3K-AKT signaling and play important roles in the balance of cell metabolism and proliferation [50]. We speculated that the TNF and NF-κB signaling pathways induced cell apoptosis and were activated to balance the cell metabolism and cell proliferation of oysters under growth-inhibited conditions.

### Endocytosis-associated signaling transduction plays an indispensable role in oyster growth

Endocytosis plays an essential role in controlling the activity and quantity of the membrane receptors, including the G protein-coupled receptors (GPCRs) and the receptor tyrosine kinases (RTKs), thereby increasing or decreasing receptor-mediated signaling transduction [51]. Receptors can be recycled to the plasma membrane for signal transduction or be retained in multivesicular bodies (MVBs) and translocate to the lysosomes for degradation. In the EGFR-mediated pathways, ligand binding induces endocytosis of EGFR into endosomes and eventually into the lysosomes for degradation, which reduces the number of active EGFR molecules, attenuates the signaling, and finally influences cell proliferation and individual growth [52]. In this study, the *MVB* genes, *VPS* genes, *CHMP* genes, and the *EGFR* gene were all highly expressed in slow-growth oysters, which suggested that the endocytosis-mediated lysosome degradation might inhibit the oyster growth through attenuating the growth-related signaling transduction.

Ubiquitination of downstream signaling molecules on the plasma membrane, such as the Ras GTPases, also leads to endocytosis and signal downregulation, which impacts organ development [53, 54]. We found that several E3 ubiquitin ligase genes and the genes of GTP-binding protein were significantly highly expressed under growth-inhibited conditions. Furthermore, the endocytic pathway was reported to be sensitive to nutrient availability. Endocytosis of non-essential proteins indeed may assist survival under conditions of limited energy supply. In yeast, low glucose levels stimulate the endocytosis of plasma membrane proteins to the lysosome and block their recycling to the cell surface, thereby promoting endocytic flux to the vacuole and providing energy for cell metabolism [55]. Therefore, we propose that endocytosis-induced signaling transduction, ubiquitination, and plasma membrane protein degradation processes might govern the growth of oysters by balancing the environmental input with endogenous signaling pathways.

### LncRNAs participate in transcriptional regulation of the growth-related genes

The lncRNAs are critical regulators of gene expression in growth, development, and environmental stress [56–59]. In this study, we presented a catalog of lncRNAs expressed in *C. gigas*, including 18,969 novel lncRNAs and 2396 known lncRNAs. Analysis of their target genes allowed identification of 265 DEGs-DELs *cis*-regulatory pairs and 618 DEGs-DELs *trans*-regulatory pairs that play indispensable roles in cell metabolism and proliferation. We found that the expressions of *SREBP1*/*p62*-*LNC_001270*, *CDC42*-*LNC_003322*, *CaM*-*LNC_011563*, *FAS*-*LNC_006260*, *PIK3CA*-*LNC_012905*, and *TUBA1A*-*LNC_003736* were all influenced by environmental temperature and nutrient level. Once the expression of *LNC_001270* decreased under conditions of low temperature and fasting treatment, its target gene *p62* was upregulated. As the *LNC_001270* is located at the antisense strand of its target gene, *LNC_001270* may hybridize with sense RNA as RNA duplexes or establish complex configurations as RNA–DNA duplexes and triplexes [60] to inhibit the expression of *p62*, and eventually influence the autophagy process. As a master regulator of lipid homeostasis [61], the transcription factor *SREBP1* is located downstream of the *LNC_001270*, thus we speculate that the *LNC_001270* may also participate in lipid metabolism. The *CDC42* gene, which belongs to the Rho GTPases family, had been reported to be involved in the regulation of the cell cycle, cell survival, actin cytoskeleton organization, and even membrane trafficking [56]. Recent studies found that the *CDC42* also participated in insulin synthesis, insulin granule mobilization, exocytosis-mediated insulin secretion, and the β cell proliferation [62, 63]. Regulation of *CDC42* is associated with miRNAs, lncRNAs, and even post-translational modifications [64, 65]. In this study, we identified *LNC_003322* from the upstream of *CDC42* whose expression was under the positive regulation of *LNC_003322*. Furthermore, *LNC_011563* is located downstream of its target gene *CaM* and positively regulates the expression of *CaM* with the fluctuation of culture temperature and nutrient levels. The insulin signaling downstream key kinase *PI3KCA*, fatty acid, and cholesterol metabolism-related genes *SREBP1* and *FAS* were all altered by food abundance under negative regulation of *LNC_012905*, *LNC_001270*, and *LNC_006260*. Of all the *trans*-acting lncRNAs, the expression of *XR_9000022.2*, *LNC_008019*, *LNC_015817*, *LNC_000838*, *LNC_00839*, *LNC_011859*, *LNC_007294*, *LNC_006429*, *XR_002198885.1*, and *XR_902224.2* was closely related to the expression of genes enriched in AMPK signaling, insulin signaling, pyrimidine metabolism, calcium signaling pathway, endocytosis, apoptosis, and tyrosine metabolism pathways. The lncRNAs may serve as functional hub to play a muti-function in regulating growth-related genes in oysters.

## Conclusions

**Fig. 7:**
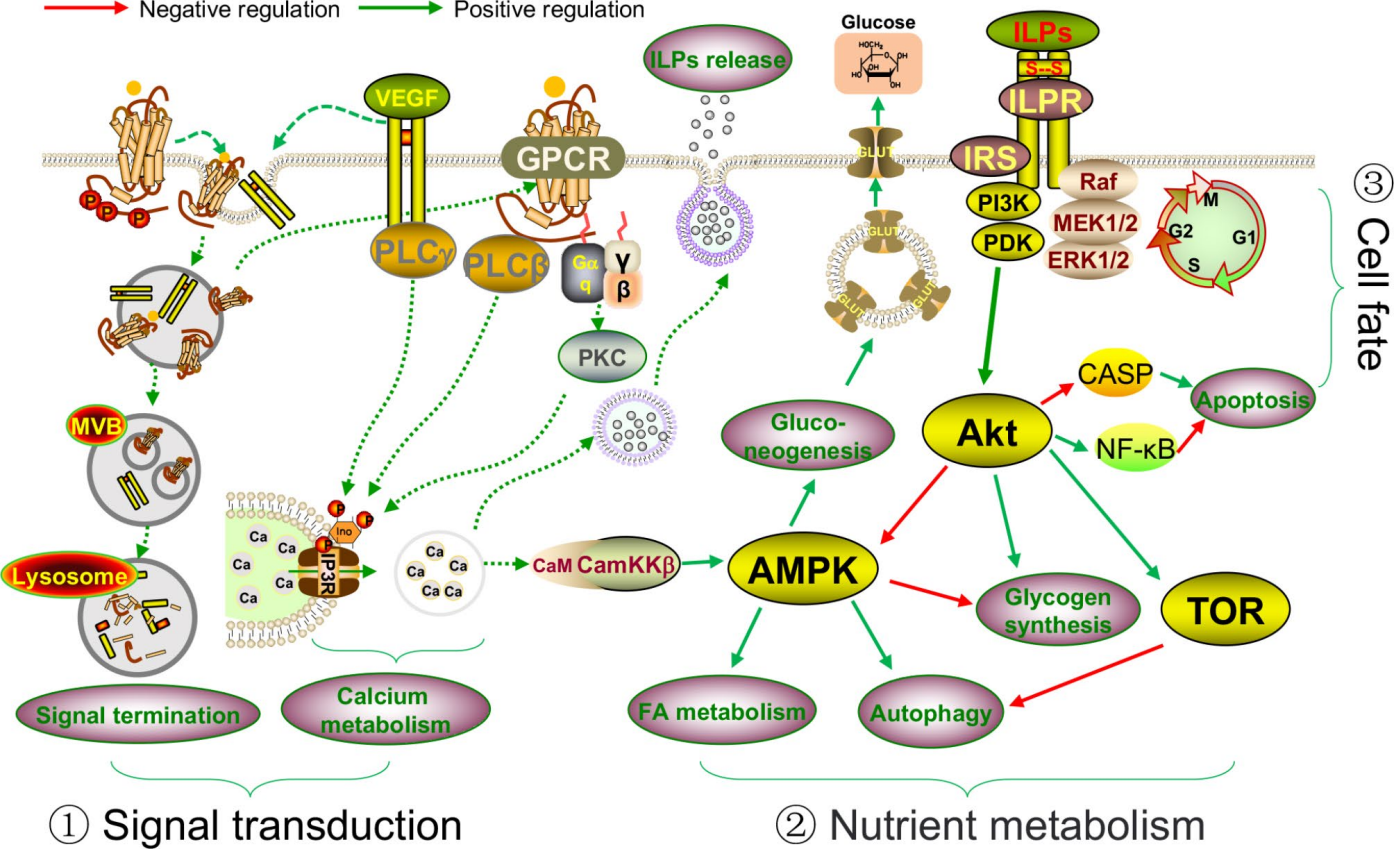
The proposed regulatory network involved in growth regulation of the oyster with the influence of environmental factors. ① In slow-growth oysters, internalization and degradation of the membrane receptor (RTKs and GPCRs) through the MVB and lysosome cause signaling termination. In normal-growth oysters, some of the GPCRs recycle to the membrane and activate the PKC and PLC, ultimately increasing calcium metabolism and insulin-like peptides (ILPs) release. Meanwhile, the secretion of ILPs is highly dependent on glucose levels, neurotransmitters, and neuropeptide activity. ② Under normal growth conditions, the ILPs and the ILPR-mediated PI3K/AKT, MPAK/ERK, and TOR signaling pathways play indispensable roles in cell and organism growth, cell cycle regulation, and cell metabolism. Under growth-inhibited conditions, activation of AMPK signaling influences fatty acid metabolism, gluconeogenesis, autophagy, and cell metabolism processes. ③ Activation of the ILPR-mediated PI3K/AKT signaling pathway also regulates the expression of *CASP* and the activity of NF-κB signaling, ultimately causing cell apoptosis or survival

In conclusion, cell metabolism, cell proliferation, and the signaling transduction processes are all important for oyster growth and are easily affected by environmental factors (Fig. 7). Under normal growth conditions, the insulin-like peptides and the ILPR-mediated pathways are active to regulate the processes related to cell metabolism and cell proliferation. In this process, several factors, including the kinase, the CaM, and the neuropeptides, all affect the activity of the insulin signaling pathway. While under growth-inhibited conditions, AMPK signaling is activated to block energy consumption, and autophagy is also activated to maintain the balance of energy metabolism. These processes are always energy-consuming and hindered the growth of oysters. In addition, the internalization of the membrane receptors such as the RTKs and GPCRs is crucial for growth-related signaling transduction. The lncRNAs could serve as functional hub to play crucial roles in the transcription regulation of growth-related genes.

## Materials and methods

### Growth-inhibited oyster model

The *C. gigas* spat with similar size (shell height around 2 mm) were initially reared at 25 °C with a 12 h: 12 h light: dark photoperiod. To build the growth-inhibited model, we randomly selected *C. gigas* spat for culture at a lower temperature (15 ℃). This slow-growth spat (SG) was used for comparative transcriptome analysis with spat cultured at a normal temperature (25 ℃, normal growth, NG). For each cultured temperature, three random replicates were allocated, and the survival, feeding situation, and shell height of the oyster spat were monitored daily. After 10 days, the shell height of the spat cultured at 25 ℃ was significantly higher (4 mm ± 0.3 mm) than that of the spat cultured at 15 ℃ (2 mm ± 0.3 mm) [43].

### RNA extraction, library construction, and sequencing

Total RNA was extracted from the soft body of normal-growth oysters and slow-growth oysters by using the Trizol reagent (Invitrogen, USA) according to the manufacturer’s instructions. The RNA purity and concentration were evaluated with the Nano Photometer^®^ spectrophotometer (IMPLEN, CA, USA) and Qubit^®^ RNA Assay Kit in Qubit^®^ 2.0 Fluorometer (Life Technologies, CA, USA). The RNA integrity was assessed with the RNA Nano 6000 Assay Kit of the Bioanalyzer 2100 system (Agilent Technologies, CA, USA). For library construction, a total amount of 20 ng RNA per sample was used as input. Firstly, ribosomal RNA was removed with the Epicentre Ribo-zero™ rRNA Removal Kit (Epicentre, USA), and the rRNA-free residue was cleaned up by ethanol precipitation. Subsequently, sequencing libraries were generated using the rRNA-depleted RNA by using NEBNext^®^ Ultra™ Directional RNA Library Prep Kit for Illumina^®^ (NEB, USA) following the manufacturer’s recommendations. Briefly, fragmentation was carried out using divalent cations under elevated temperature in NEBNext First Strand Synthesis Reaction Buffer (5X). First-strand cDNA was synthesized using a random hexamer primer and M-MuLV Reverse Transcriptase (RNase H-). Second-strand cDNA synthesis was performed using DNA polymerase I and RNase H. In the reaction buffer, dTTPs were replaced by dUTP. The remaining overhangs were converted into blunt ends via exonuclease/polymerase activities. After adenylation of the 3’ ends of the DNA fragments, a NEBNext adaptor with a hairpin loop structure was ligated to prepare for hybridization. To select cDNA fragments of 150 ~ 200 bp in length, the library fragments were purified with the AMPure XP system (Beckman Coulter, Beverly, USA). Thereafter, 3 µl of USER Enzyme (NEB, USA) was incubated at 37° C for 15 min, followed by 5 min at 95 °C before proceeding to PCR. The PCR reaction was performed with Phusion High-Fidelity DNA polymerase over 15 cycles. The library quality was assessed on the Agilent Bioanalyzer 2100 system. Finally, the product was performed on a cBot Cluster Generation System using the TruSeq PE Cluster Kit v3-cBot-HS (Illumina) according to the manufacturer’s instructions. After cluster generation, the libraries were sequenced on an Illumina Hiseq 2500 platform for 150 bp paired-end reads.

### Quality control and transcriptome assembly

Clean reads were obtained after trimming the reads with adapters, low quality, and uncertain ‘N’ with the ratio of ‘N’ > 10% using fastp [66]. Reference genome and gene model annotation files were downloaded from the genome website directly (RefSeq: GCF_902806645.1, https://ftp.ncbi.nlm.nih.gov/genomes/refseq/invertebrate/Crassostrea_gigas/latest_assembly_versions/GCF_902806645.1_cgigas_uk_roslin_v1/). An index of the reference genome was built, and paired-end clean reads were aligned to the reference genome using HISAT2 (v2.0.4). The HISAT2 was run with “--rna-strandedness RF”, and other parameters were set as default [67]. The mapped reads of each sample were assembled with StringTie (v2.1.1) [68] in a reference-based approach. Transcripts from all samples were then merged together with StringTie merge mode to build a consensus set of transcripts across samples. Transcript abundances were estimated and read coverages were generated using StringTie. Then, the assembled transcripts were compared to the known genes recorded in the database using Cufflinks compare [69]. The unknown transcripts were used for further lncRNA analysis.

### LncRNAs identification

The pipeline of novel lncRNAs prediction was as follows: firstly, the unknown transcripts were selected for identification of lncRNAs, including those exonic overlap on the opposite strand (“x”), those fully contained within a reference intron (“i”), and those located in intergenic regions (“u”). Then, transcripts with the exon number ≥ 2, length > 200 bp, and FPKM ≥ 0.5 were chosen, and the transcripts that overlapped with known protein-coding genes on the same strand were discarded. Finally, the CNCI (Coding-Non-Coding-Index) (v2), CPC (v0.1) (--e value le-10), and PFAM (Pfam-scan) (v1.3) (--E 0.001; --domE 0.001; --pfamB) [70–72] were used to filter transcripts with coding potential. The remaining transcripts were considered reliable lncRNAs. Furthermore, lncRNAs and mRNAs were compared and analyzed in terms of structure and sequence characteristics, including the transcript length, exon number, and predicted ORF length. The ORF sequence of mRNA was extracted by the annotation of known gene structure, and the ORF sequence of lncRNA was predicted by EMBOSS: getorf. The ORF sequences were converted into protein sequences, and then the length distribution was obtained.

### Identification and functional annotation of differentially expressed genes

The differential expression analysis was carried out using the R package DESeq (1.18.0), and the transcripts with *P*-adjust < 0.05 and | log_2_ (fold change) | >1 were assigned as differentially expressed genes [73]. Furthermore, GO and KEGG enrichment analysis of DEGs were performed with ClusterProfiler (v4.1.4) [74–77]. The GO terms and KEGG pathways with a corrected *P-value <* 0.05 were considered significantly enriched. After enrichment, all the significantly enriched GO terms were clustered based on semantic similarity by using the R package rrvgo (threshold = 0.7) [78].

### Prediction of target genes

To identify *cis*-acting lncRNAs that can regulate neighboring coding genes, coding genes within the regions 10 kb upstream or downstream of the DELs were selected. The prediction of *trans*-target genes was based on the co-expression relationship between DEGs and DELs. We calculated the Pearson correlation coefficients (r) using the R function “cor. test” and selected the DEGs-DELs pairs with | r |>0.99 for further analysis. Finally, the DEGs-DELs interaction networks were constructed and visualized with Cytoscape software [79].

#### Fasting and re-feeding experiment

For the fasting and re-feeding experiment, ninety 8-month-old *C. gigas* were randomly divided into three groups and starved for 14 days, then refed with frozen Chlorella ad libitum. Samples were collected before fasting, on day 5 and day 14 during fasting, and 1 h, and 6 h after re-feeding, respectively. At each time point, eight tissues including the labial palp, gill, mantle, digestive gland, hematocyte, heart, visceral ganglia, and adductor muscle from six oysters were rapidly excised and frozen in liquid nitrogen for further RNA extraction and cDNA synthesis. Furthermore, the cDNA used for real-time PCR analysis was derived from a mixture of the eight tissues at each sampling point consisting of an equal amount of each tissue.

### qRT-PCR analysis

To validate the transcriptome regulatory relationship between the DEGs and DELs, cDNA from the temperature treatment and fasting/re-feeding treatment samples were all used for qRT-PCR. The primer sets were designed using Primer Express software (Applied Biosystems, USA), and the sequences were shown in Additional file 1: Table S10. All real-time PCRs were carried out in a LightCycler 480 real-time PCR machine (Roche, Switzerland) with a mixture of 5 µL 2×SYBR Premix ExTaq (Qiagen, Germany), 1.0 µL of diluted cDNA, 3 µL of PCR-grade water, and 0.5 µL of each 10 µM primer. The PCR was initiated by denaturation at 95 °C for 30 s; followed by 40 amplification cycles at 95 °C for 15 s and 60 °C for 30 s. Dissociation protocols were used to measure the melting curves. The relative expression level was calculated with the 2^−ΔΔCt^ method [80], and the data were expressed as the mean ± SD. Statistical significance was determined by the one-way ANOVA and Student’s *t*-test for multiple groups and two groups comparison, and *P < 0.05* was considered statistically significant.

### Electronic supplementary material

Below is the link to the electronic supplementary material.

Supplementary Material 1

Supplementary Material 2

Supplementary Material 3

Supplementary Material 4

## Data Availability

All raw RNA-Seq data have been deposited in the NCBI Sequence Read Archive with BioProject accession no. PRJNA951908 (sequence accessions: SRR24058831-SRR24058836, https://www.ncbi.nlm.nih.gov/search/all/?term=PRJNA951908).

## Abbreviations

KEGG: Kyoto encyclopedia of genes and genomes
GO: Gene ontology
DEGs: Differentially expressed genes
DELs: Differentially expressed lncRNAs
NG: Normal-growth oysters
SG: Slow-growth oysters
LncRNAs: Long non-coding RNAs
ILPs: Insulin-like peptides
*HMG-CoA*: *3-hydroxy-3-methylglutaryl-coenzyme A reductase*
*HSL*: *Hormone-sensitive lipase*
*SCD*: *Acyl-CoA desaturase*
*FAS*: *Fatty acid synthase*
*ILPR*: *Insulin-like peptide receptor*
*PI3KCA*: *Phosphatidylinositol 4,5-bisphosphate 3-kinase catalytic subunit alpha*
*S6Kβ1*: *Ribosomal protein S6 kinase beta-1*
*RAPTOR*: *Regulatory-associated protein of mTOR-like*
*CaM*: *Calmodulin*
*5HTR4*: *5-hydroxytryptamine receptor 4*
*α1-ARs*: *Alpha-1 A adrenergic receptor*
*AChR*: *Acetylcholine receptor subunit alpha-like*
*NMUR2*: *Neuromedin-U receptor 2*
*CCKR*: *Cholecystokinin receptor type A*
*MTMR14*: *Myotubularin-related protein 14*
*ATG*: *Autophagy-related protein 101*
*LC3*: *Microtubule-associated proteins 1 A/1B light chain 3 A*
*p62*: *Sequestosome-1*
*HSP*: *Heat shock protein*
*CHMP*: *Charged multivesicular body protein*
*VPS*: *Vacuolar protein sorting-associated protein*
*BIRC*: *Baculoviral IAP repeat-containing protein*
*CASP*: *Caspase*
*IAPs*: *Inhibitor of apoptosis*
*BCL2L2*: *BCL-2-like protein 2*
*TRAF2*: *TNF receptor-associated factor 2*
*XIAP*: *E3 ubiquitin-protein ligase XIAP*
