# Supplementary material for: Integrated analysis of mRNAs and lncRNAs reveals candidate marker genes and potential hub lncRNAs associated with growth regulation of the Pacific Oyster, Crassostrea gigas
Source: BMC Genomics. 2023 Aug 10;24:453. doi: 10.1186/s12864-023-09543-7 (PMC10416452; doi:10.1186/s12864-023-09543-7)
Supplement: Supplementary file 4 — Supplementary Material 4 [file 12864_2023_9543_MOESM4_ESM.docx]

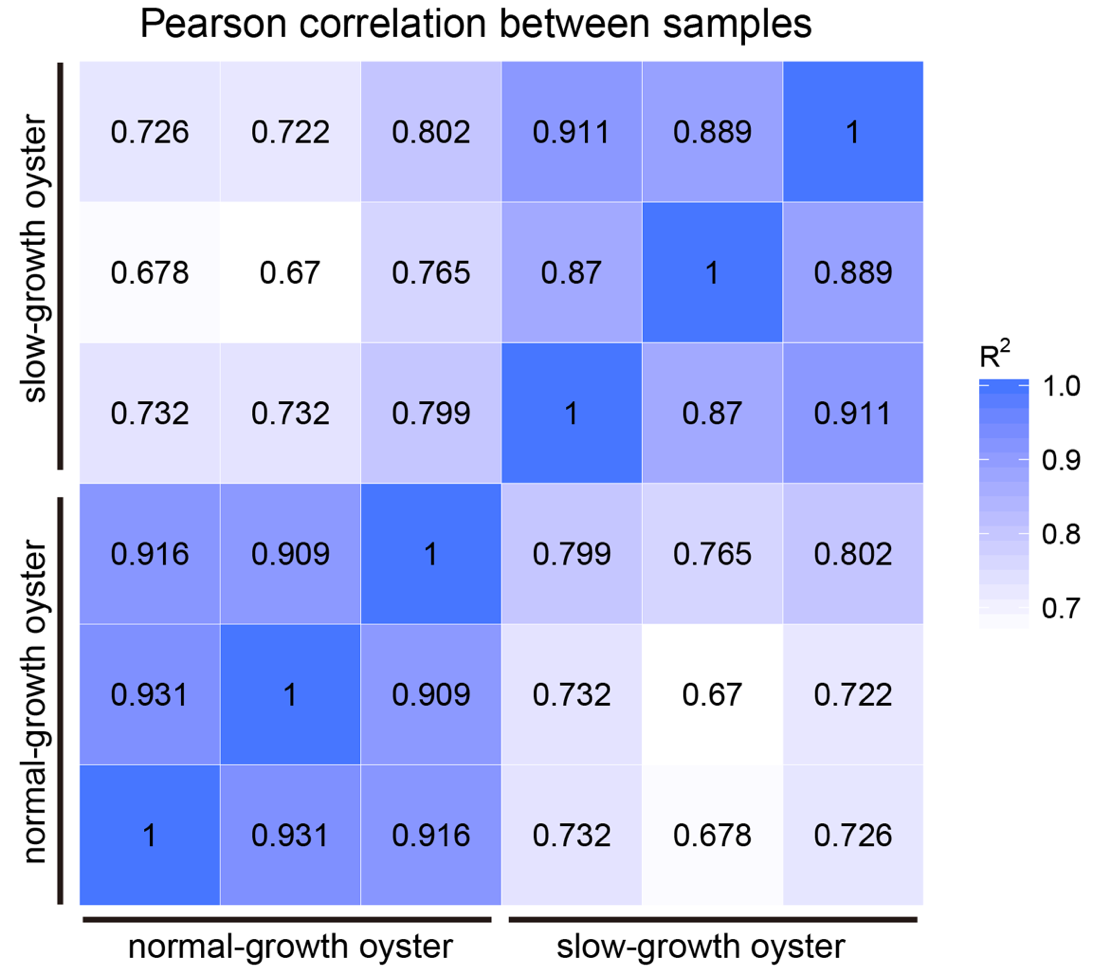

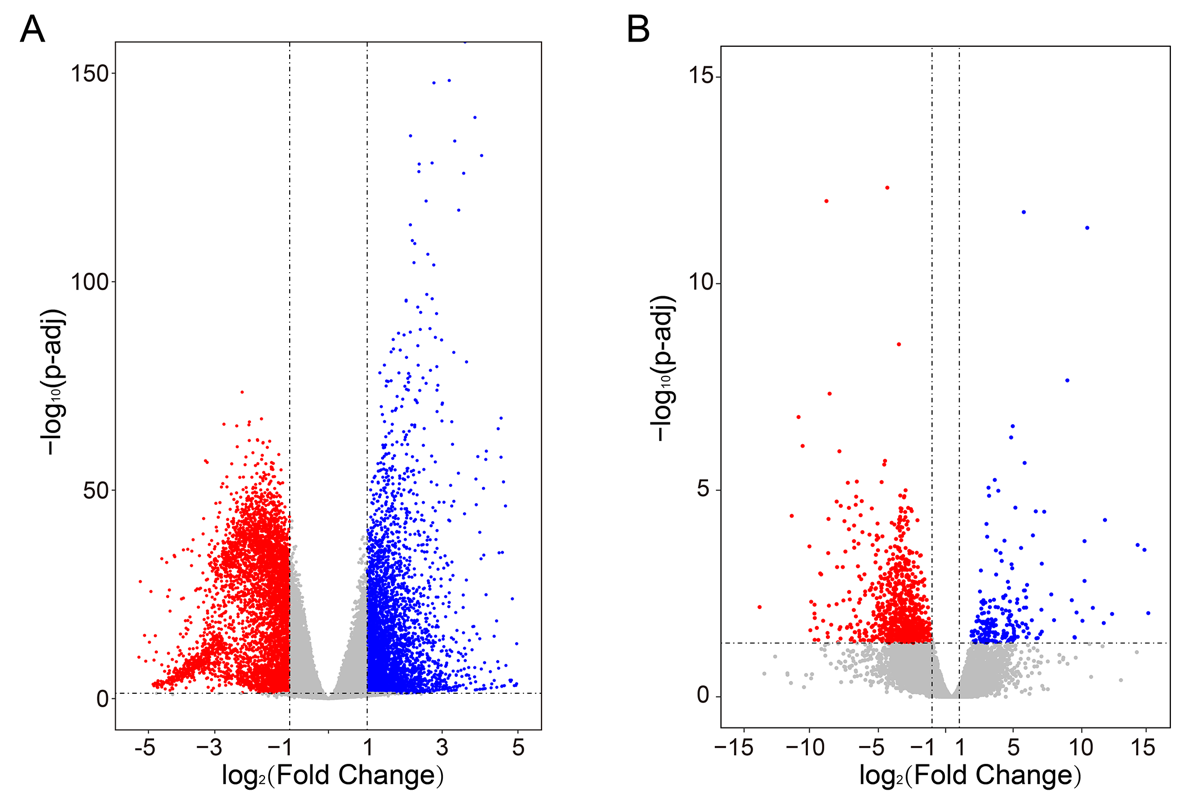
**Fig. S1** Correlation analysis of gene expression among biological replicates in the normal- and slow-growth oysters.

**Fig. S2** Differentially expressed genes (DEGs) and lncRNAs (DELs) between normal-growth oysters and slow-growth oysters. (A) The volcano plot of DEGs between the normal- and slow-growth oysters (B) The volcano plot of DELs between the normal- and slow-growth oysters. Red represented the genes and lncRNAs highly expressed in the slow-growth oysters, blue represented the genes and lncRNAs highly expressed in the normal-growth oysters, and grey represented genes and lncRNAs with no significant difference in the normal- and slow-growth oysters.
